# [Retracted] MicroRNA-1180 is associated with growth and apoptosis in prostate cancer via TNF receptor associated factor 1 expression regulation and nuclear factor-κB signaling pathway activation

**DOI:** 10.3892/ol.2025.15253

**Published:** 2025-09-01

**Authors:** Deyuan Zhu, Wenxi Gao, Zhongmin Zhang

Oncol Lett 15: 4775–4780, 2018; DOI: 10.3892/ol.2018.7914

Following the publication of this paper, it was drawn to the Editor's attention by a concerned reader that the ‘Control’ and ‘miR-NC’ data panels in Fig. 2B, showing the results of cell invasion assay experiments, were overlapping, suggesting that these data had been derived from the same original source, even though the results from differently performed experiments were intended to have been portrayed. An independent analysis of the data in this paper in the Editorial Office subsequently revealed that the ‘Control’ and ‘miR-NC’ data panels showing the results of scratch-wound assay data in Fig. 2A were also overlapping; western blots in Figs. 2 and 5 appeared to have been duplicated, even though one of the gel slices appeared to have been horizontally flipped compared with the other; and finally, control western blots featured in Fig. 3 were strikingly similar to data that subsequently appeared in a paper written by different authors at different research institutes in the journal *Open Medicine*. Finally, another concerned reader drew the Editor's attention to further overlaps of data in the western blots in Figs. 2B, 3B and 5B, including the apparently non-continuous presentation of certain of the control GAPDH western blots in these figures.

Given the large number of anomalies that have been identified with the assembly of the data in various of the figures in this paper, the Editor of *Oncology Letters* has decided that this paper should be retracted from the Journal on account of a lack of confidence in the presented data. The authors were asked for an explanation to account for these concerns, but the Editorial Office did not receive a reply. The Editor apologizes to the readership for any inconvenience caused.

